# MGMT is frequently inactivated in pancreatic NET-G2 and is associated with the therapeutic activity of STZ-based regimens

**DOI:** 10.1038/s41598-023-34666-y

**Published:** 2023-05-09

**Authors:** Kohei Yagi, Hiroaki Ono, Atsushi Kudo, Yuko Kinowaki, Daisuke Asano, Shuichi Watanabe, Yoshiya Ishikawa, Hiroki Ueda, Keiichi Akahoshi, Shinji Tanaka, Minoru Tanabe

**Affiliations:** 1 Department of Hepatobiliary and Pancreatic Surgery, Graduate School of Medicine, Tokyo Medical and Dental University, 1-5-45 Yushima, Bunkyo-Ku, Tokyo, 113-8519 Japan; 2 Department of Hepatobiliary and Pancreatic Surgery, Tokyo Medical and Dental University, Tokyo, Japan; 3 Department of Comprehensive Pathology, Graduate School of Medical and Dental Sciences, Tokyo Medical and Dental University, Tokyo, Japan; 4 Department of Molecular Oncology, Graduate School of Medicine, Tokyo Medical and Dental University, Tokyo, Japan

**Keywords:** Neuroendocrine cancer, Molecular medicine

## Abstract

O6-methylguanine-DNA methyltransferase (MGMT) has been linked with alkylating agent resistance and tumor growth suppression. However, its role remains undetermined in pancreatic neuroendocrine tumors (Pan-NET). The MGMT expression was examined by immunohistochemistry in 142 patients to evaluate MGMT immunoreactivity and clinicopathological factors. We analyzed the relationship between MGMT expression and treatment efficacy in 19 patients who received STZ-based regimens. In 142 Pan-NET, 97 cases (68.3%) were judged as MGMT-positive and 45 cases (31.6%) as negative. MGMT negativity was significantly more common in NET-G2 (62.5%) than in NET-G1 (11.2%, *p* < 0.001). MGMT-negative cases were associated significantly with larger tumor size (*p* < 0.01), higher Ki-67 index (*p* < 0.01), higher mitotic index (*p* < 0.05), and more frequent liver metastasis (*p* < 0.05). Of the 19 cases treated with STZ, 6 cases were determined as SD and 4 cases as PD in MGMT-positive patients (N = 10), while 5 cases were determined as PR and 4 cases as SD in MGMT-negative patients (N = 9). Progression-free survival in MGMT-negative cases was significantly better than in MGMT-positive cases (*p* < 0.05). MGMT expression was lower in NET-G2 than in NET-G1, and STZ-based regimens improved the therapeutic outcomes of MGMT-negative Pan-NET. These findings indicate that NET-G2 may represent a better therapeutic target for STZ treatment.

## Introduction

Pancreatic neuroendocrine neoplasms (Pan-NENs) are tumors arising from pancreatic endocrine cells^1^ and considered to be clinically rare; however, the incidence of these tumors has recently been increasing^2–4^. Disease outcomes are categorized based on the tumor grade defined as NET-G1/G2/G3, and NEC-G3 based on the 2017 World Health Organization (WHO) classification.

Metastases to distant organs are often present when a diagnosis of Pan-NENs is confirmed^5^. Whereas surgical resection is the curative treatment for patients with Pan-NENs systemic chemotherapy is indicated for surgically unresectable cases. There are various established agents for the treatment of Pan-NENs, and the decision for systemic therapy is based on biological factors such as the tumor burden, grade, and growth rate^6^.

Recently, molecular-targeted agents have been often used in unresectable well-differentiated pancreatic neuroendocrine tumors (Pan-NET). Clinical administration of sunitinib and everolimus is indicated exclusively for advanced low-grade Pan-NET^9–11^. On the other hand, chemotherapy with streptozocin (STZ), an alkylating agent, has been reported to be effective, especially in patients with a Ki-67 index greater than 5%^7^. It is usually used in combination with other drugs, such as 5-fluorouracil or doxorubicin^8^. Thus, STZ plays an essential role in the treatment of locally advanced or distant metastatic Pan-NET.

The mechanism of action of alkylating agents involves alkylation of the O6-guanine moiety of DNA to produce O6-methylguanine (O6MeG), which mismatches with thymine and activates the mismatch repair mechanism, while subsequent DNA double-strand breaks induce apoptosis^9^. MGMT is known to inhibit the action of alkylating agents through dealkylation of DNA^10^. Therefore, decreased MGMT activity, such as with reduced MGMT protein expression or methylation of MGMT, which is often observed in cancer cells, may increase the drug sensitivity and contribute to the antitumor properties of alkylating agents^10^.

Temozolomide (TMZ), an alkylating agent, is a key drug for the treatment of glioblastoma. The relationship between methylation of MGMT and the therapeutic efficacy of temozolomide has been studied extensively in clinical cases of glioblastoma^11^. Since MGMT methylation has been shown to be a predictor of the therapeutic response to temozolomide^12–14^, epigenetic regulation may be crucial in brain tumors^11^.

Immunohistochemistry (IHC) is also used to analyze MGMT protein expression. In glioblastoma, IHC is useful as a diagnostic histopathology test, and MGMT levels are assessed by the percentage of MGMT-positive cells in the nucleus^15^. However, some issues have limited the usefulness of clinical tests for evaluating MGMT expression, such as the sensitivity of MGMT positivity because of the cutoff used in immunostaining.

Several reports related to the effectiveness of alkylating agents have also been documented, especially for temozolomide and even in Pan-NET. However, the relationship between MGMT expression and STZ treatment efficacy has not been elucidated^16–26^.

In this study, we examined MGMT expression by immunohistochemistry staining in surgically resected Pan-NET. We also tried to determine its impact on the therapeutic efficacy of STZ treatment.

## Methods

### Patient and methods

This retrospective study included a total of 392 patients who were histologically diagnosed with Pan-NENs between November 2002 and December 2020 at Tokyo Medical and Dental University. The use of resected samples and experimental protocol of this study were approved by the Human Ethics Review Committee of the Faculty of Medicine in Tokyo Medical and Dental University (permission No. M2000-1080), written informed consent to have data from their medical records used in research was obtained from all patients. Patients were anonymously coded in accordance with ethical guidelines, as set out in the Declaration of Helsinki. Among these patients, 19 patients were treated with STZ; 2 patients received STZ monotherapy and 17 patients received a combination of STZ and S-1, as previously described^8^. The clinical response of the 19 STZ-treated patients was evaluated based on the Response Evaluation Criteria in Solid Tumors (RECIST version 1.1) by comparing CT or MRI images before and after treatment.

### Immunohistochemistry

All 142 Pan-NET samples were obtained during surgery from patients treated at Tokyo Medical and Dental University between Feb 2004 and Dec 2019 and prepared for MGMT staining for immunohistochemistry (IHC) staining by surgical resection or as biopsy specimens. Anti-MGMT antibody (Clone MT 3.1, #MS-470-P1) was from Themo Scientific (Fremont, CA, USA). Non-neoplastic cells such as endothelial cells and islets of Langerhans in tumor samples were used as internal positive controls. MGMT expression was considered positive if nuclear staining was observed in more than 10% of the tumor cells, as previously reported^16^. MGMT expression was independently evaluated and pathologically assessed in all surgically resected lesions by two investigators (KY and YK). In cases of disagreement, the MGMT status was determined by consensus after discussion between the two observers.

### Statistical analysis

Comparisons between groups were made by Fisher’s exact test or Mann–Whitney U test. Variables with a *p* value < 0.05 were incorporated into a multivariate analysis. The multivariate analysis used a logistic regression model to examine the factors associated with a poor prognosis. Survival curves were constructed by the Kaplan–Meier method and compared with the log-rank test as necessary. Progression-free survival (PFS) was defined as the period from the start of treatment to the appearance of progressive disease or death. All statistical analyses were performed using SPSS version 21 software (IBM, Tokyo, Japan). A *p* value < 0.05 was considered statistically significant**.**

## Results

**Figure 1:**
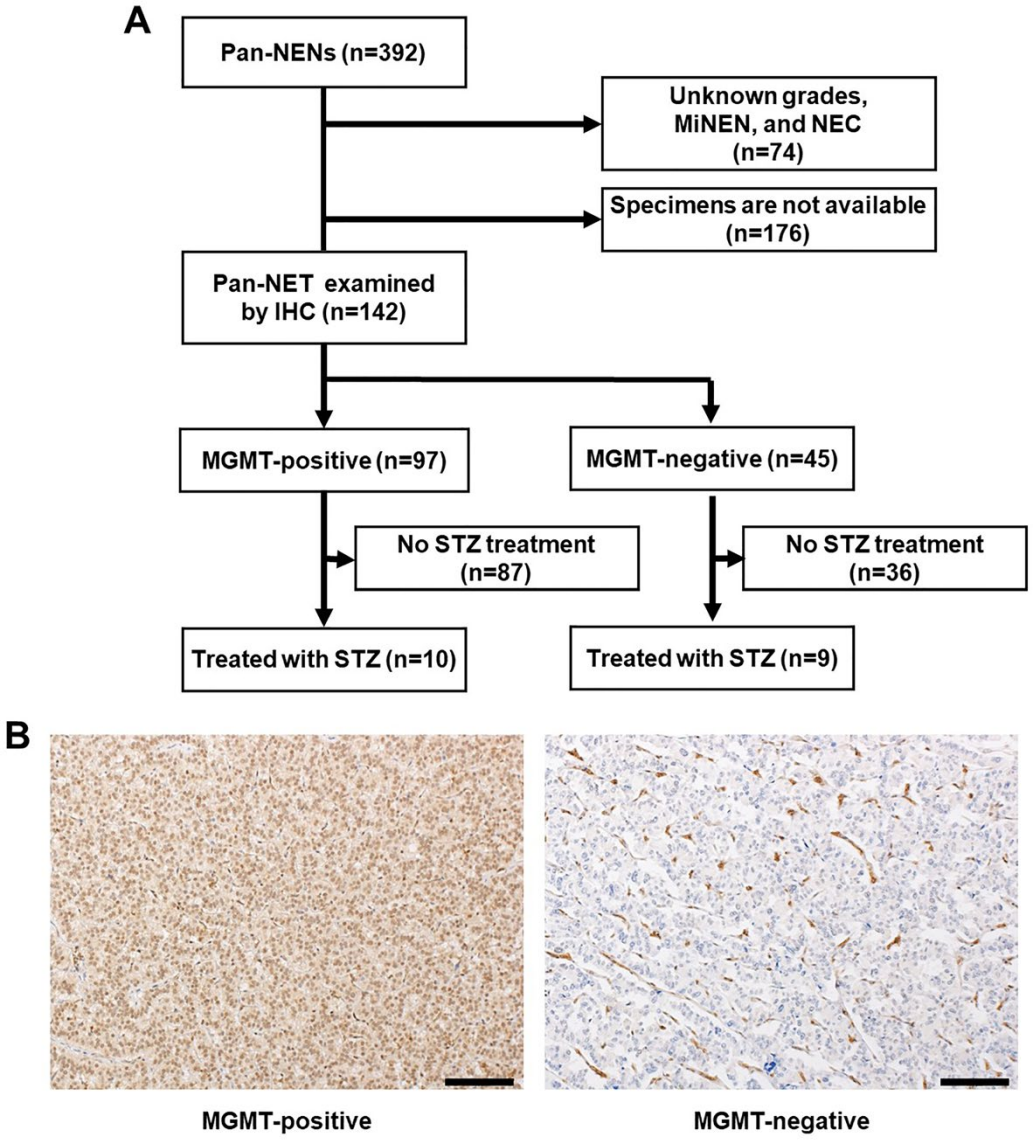
Study design and representative images of MGMT expression in Pan-NENs. (**A**) Design of this study. Pan-NET patients (N = 142) were enrolled and evaluated for MGMT expression by IHC staining. (**B**) Representative immunohistochemical staining of positive and negative MGMT expression. Scale bar indicates 40 μm.

A total of 392 cases diagnosed as Pan-NENs were enrolled in this study. Of these, 65 patients with tumors of unknown grade, 5 patients with MiNEN, and 4 patients with NEC were excluded from the entire cohort. In addition, no specimens were available for 176 patients because most of the lesions were in an advanced stage or an early stage with small diameters, making it difficult to obtain specimens for pathology (Fig. 1A).

Among these patients, MGMT expression was examined by IHC staining in 142 Pan-NET. Among the samples, 141 were collected by surgery, and the remaining sample was collected by fine-needle aspiration (FNA). The operable lesions were mostly in early stages. In total, 135 patients underwent resection of primary lesions, and the remaining patients with advanced lesions underwent resection of liver metastases (Table 1). MGMT was positive in 99 of 146 cases (67.8%) (Fig. 1A). Representative staining of positive and negative MGMT staining images are shown in Fig. 1B.

**Table 1:** Clinicopathological factors of 142 patients with Pan-NET.

Characteristics	Total n = 142
Age, median (range)	57.5 (18–80)
Sex, male/female	72/70
Genetic syndrome, n (%)	12 (8.5)
MEN type 1	10
VHL	2
Lymphnode metastasis, n (%)	25 (17.6)
Liver metastasis, n (%)	41 (28.9)
Synchronous	27 (19.0)
Metachronous	14 (9.9)
Ki-67 index, median (range)	1.9 (0.07–33.5)
Mitosis, per 10HPF, median (range)	1 (0–25)
Tumor grade	
NET-G1	80
NET-G2	56
NET-G3	6
Specimen, origin	
Primary lesion (pancreas)	135
Metastatic lesion (liver)	7
Specimen, procedure	
Operation	141
Endoscopy (FNA)	1
Surgical procedue	
Pancreatectomy	116
Hepatectomy ± pancreatectomy	26

The patient backgrounds of MGMT positive and negative expressions are shown in Table 2. There were no significant differences in age, sex, genetic syndrome, lymph node metastasis, or expression of neuroendocrine tumor markers such as chromogranin A, synaptophysin, and CD-56. However, when tumor factors were considered, the diameter of the tumor in the MGMT-positive group was 22.9 mm. On the other hand, in the MGMT-negative group, the tumor diameter was 42.1 mm, which was significantly larger compared with the MGMT-positive group. Similarly, the negative group demonstrated a higher Ki-67 index and a higher mitosis index (Ki-67 index, 4.0 for positive MGMT vs 7.5 for negative MGMT; mitosis index, 1.2 for positive MGMT vs 2.7 for negative MGMT, respectively). Liver metastasis was significantly more frequent in the MGMT-negative group. In terms of tumor grade, the 97 MGMT-positive cases included 71 NET-G1 cases, 21 NET-G2 cases, and 5 NET-G3 cases. The 45 MGMT-negative cases included 9 NET-G1 cases, 35 NET-G2 cases, and 1 NET-G3 case. Of note, the frequency of MGMT negativity was significantly higher in NET-G2 (35/56 cases, 62.5%) than in NET-G1 (9/80 cases, 11.2%, *p* < 0.001).

**Table 2:** Clinicopathological factors with MGMT expression levels.

Clinicopathological factor (n = 142)	MGMT-positive (n = 97)	MGMT-negative (n = 45)	*p* value
Clinical factor			
Age, years, median (range)	59 (18–79)	56 (28–80)	0.97
Sex, male, n (%)	47 (48)	27 (57)	0.2
Genetic syndrome, n (%)			
MEN type 1	6 (6)	4 (9)	0.56
VHL	1 (1)	1 (2)	0.58
Tumor factor			
Tumor size, mean ± SD, mm	22.9 ± 24.6	42.1 ± 34.7	0.0015*
Ki-67 index, mean ± SD	4.0 ± 6.5	7.5 ± 6.5	0.0035*
Mitosis, 10 HPF, mean ± SD	1.2 ± 2.7	2.7 ± 4.1	0.036*
Lymph node metastasis, n (%)	16 (16)	8 (18)	0.85
Liver metastasis, n (%)	22 (23)	19 (42)	0.017*
Chromogranin A positive, n (%)	90 (93)	41 (91)	0.99
Synaptophysin positive, n (%)	95 (98)	44 (98)	0.99
CD-56 positive, n (%)	88 (91)	43 (96)	0.78
Functionality, nonfunctioning, n (%)	81 (84)	40 (89)	0.75
Tumor grade, n (%)			
NET-G1	71 (73)	9 (19)	< 0.001*
NET-G2	21 (22)	35 (74)	
NET-G3	5 (5)	1 (2)	

*HPF* high-power fields, *MEN* multiple endocrine neoplasia, *MGMT* O6-methylguanine DNA methyltransferase, *NEC* neuroendocrine carcinoma, *NET* neuroendocrine tumor, *VHL* von Hippel–Lindau disease.

*p* < 0.05 is considered significant.

The asterisk denotes a statistically significant difference between positive and negative MGMT expression below the 0.05 level.

**Figure 2:**
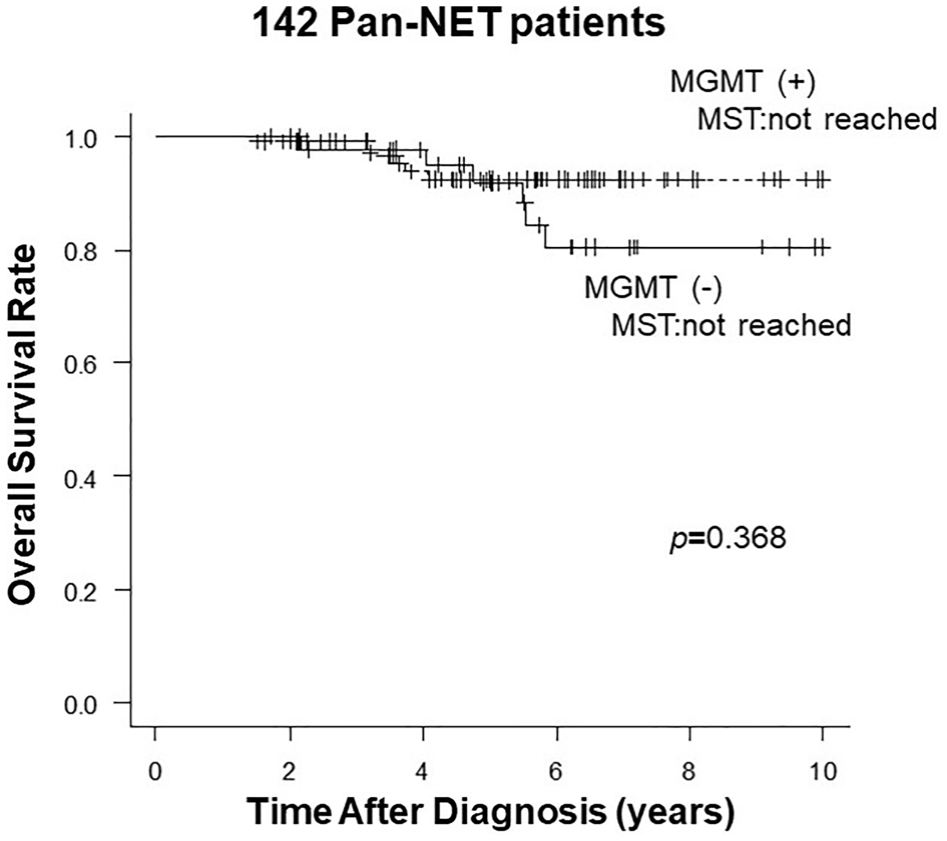
Overall survival of 142 Pan-NET patients comparing with MGMT expression levels.

In relation to MGMT expression, the prognosis tended to be worse in MGMT-negative patients, although there was no significant difference in OS from the time of diagnosis (*p* = 0.368, Fig. 2). When we examined the clinicopathological factors associated with OS, the presence of two or more mitoses, lymph node metastasis, and liver metastasis were identified as risk factors in univariate analysis (Supplementary Table 1). In multivariate analysis, only the presence of two or more mitoses was predictive of OS (hazard ratio = 5.4, *p* = 0.005).

Of the 142 patients whose tumors were evaluated for MGMT expression, 19 were treated with STZ-based regimens, 2 received STZ monotherapy, and 17 received a combination of STZ and S-1. Patients received STZ a median of 8.0 months after surgery. STZ treatment was administered in the second line in six patients, third line in six patients, and fourth line or later in seven patients (Table 3). Seventeen patients had recurrent or metastatic disease, and surgical samples were obtained prior to STZ treatment. Two patients had advanced Pan-NET with concurrent unresectable distant metastases, and surgical samples were obtained via conversion surgery after STZ treatment.

**Table 3:** Clinicopathological factors of 19 patients with Pan-NET who underwent evaluations of MGMT expression before STZ-based treatment.

Characteristics	Total n = 19
Age, years, median (range)	52 (27–75)
Sex, male/female	9/10
Genetic syndrome	
MEN type 1	0
VHL	0
Tumor factor	
Tumor size, mean ± SD, mm	56.0 ± 31.0
Ki-67 index, mean ± SD	14.6 ± 16.2
Mitosis, 10 HPF, mean ± SD	4.4 ± 17.0
Chromogranin A positive	13
Synaptophysin positive	18
CD-56 positive	17
Lymph node metastasis	7
Liver metastasis	17
Synchronous	13
Metachronous	4
Functionality, nonfunctioning	17
Tumor grade	
NET-G2	15
NET-G3	4
STZ treatment	
Month on treatment, median (range)	8.0 (0.8–31.7)
STZ treatment line	
2nd	6
3rd	6
4th or later	7

*HPF* high-power fields, *MEN* multiple endocrine neoplasia, *MGMT* O6-methylguanine *DNA* methyltransferase, *NET* neuroendocrine tumor, *STZ* streptozocin, *VHL* von Hippel–Lindau disease.

**Figure 3:**
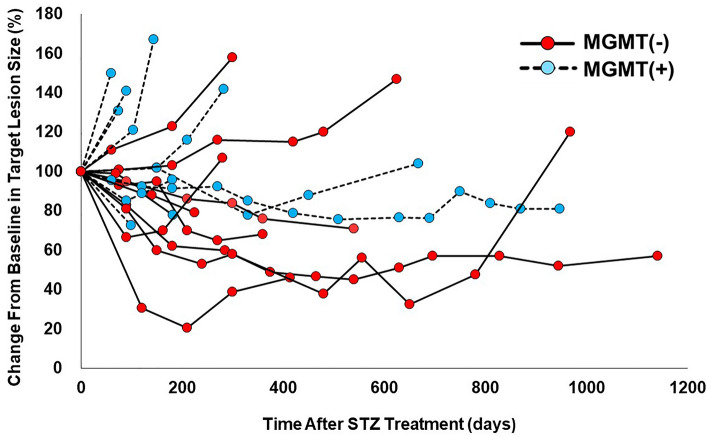
Time courses of target tumor size in each STZ-treated patient with positive and negative MGMT expression.

**Figure 4:**
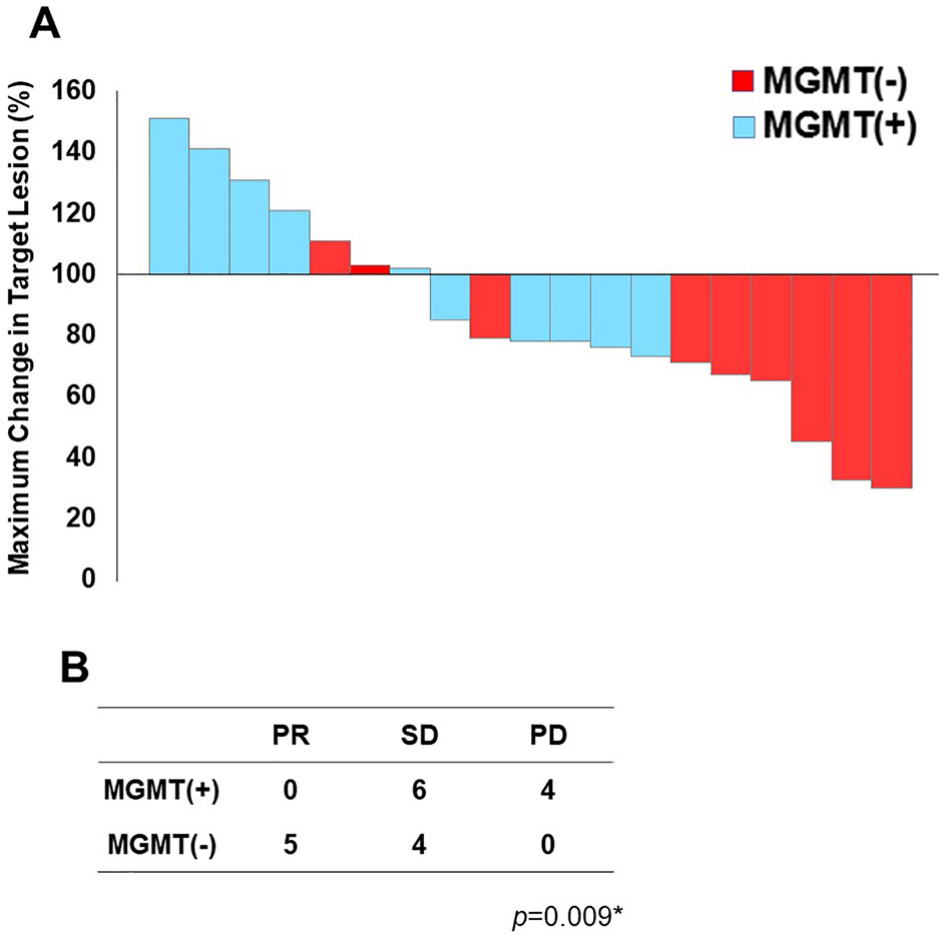
Tumor shrinkage rates after STZ administration. (**A**) The maximum shrinkage rate of the target lesion in each STZ-treated patient with positive and negative MGMT expression. (**B**) The maximum shrinkage effect as judged by RECIST criteria in patients with positive and negative MGMT expression. Maximum shrinkage rate (%) = [(sum of tumor diameters at maximum reduction − baseline diameters)/baseline diameters] × 100 for SD or PR patients. Maximum shrinkage rate (%) = [(sum of tumor diameters at maximum increase − baseline diameters)/baseline diameters] × 100 for PD patients. Statistical significance was determined by Pearson's chi-square test.

In terms of MGMT expression in relation to STZ treatment, 10 cases were MGMT positive, and 9 cases were MGMT negative (Fig. 1A). The clinicopathological background was not significantly different between negative and positive MGMT expression for STZ treatment (Table 4). In Figs. 3, 4 cases (40%) of MGMT-positive cases had early progressive disease (PD) within 100 days. However, most MGMT-negative cases showed a good treatment response including 5 (55.6%) cases with long-term disease control of more than 1 year. As shown in Fig. 4A, 6 cases showed stable disease (SD) and 4 cases were PD among 10 MGMT-positive cases treated with STZ. On the other hand, among the 9 MGMT-negative, 5 cases were classified as partial response (PR) and 4 cases as SD, indicating that the therapeutic effect was significantly better in MGMT-negative patients (*p* = 0.009) (Fig. 4B).

**Table 4:** Clinicopathological factors of STZ based treated-19 patients associated with MGMT expression levels.

Clinicopathological factor (n = 19)	MGMT-positive (n = 10)	MGMT-negative (n = 9)	*p* value
Clinical factor			
Age, years, median (range)	46 (27–65)	55 (40–75)	0.21
Sex, male	3	6	0.13
Genetic syndrome			
MEN type 1	0	0	–
VHL	0	0	–
Tumor factor			
Tumor size, mean ± SD, mm	55.7 ± 34.2	56.2 ± 20.7	0.6
Ki-67 index, mean ± SD	16.4 ± 8.9	12.5 ± 8.7	0.4
Mitosis, 10 HPF, mean ± SD	5.4 ± 6.8	3.3 ± 3.4	0.78
Chromogranin A positive	6	7	0.63
Synaptophysin positive	9	9	0.53
CD-56 positive	9	8	0.74
Lymph node metastasis	4	3	0.57
Liver metastasis	9	8	0.94
Synchronous	6	7	0.37
Metachronous	3	1	0.31
Functionality, nonfunctioning	9	8	0.74
Tumor grade			
NET-G2	6	9	0.054
NET-G3	4	0	

*HPF* high-power fields, *MEN* multiple endocrine neoplasia, *MGMT* O6-methylguanine *DNA* methyltransferase, *NET* neuroendocrine tumor, *STZ* streptozocin, *VHL* von Hippel–Lindau disease.

**Figure 5:**
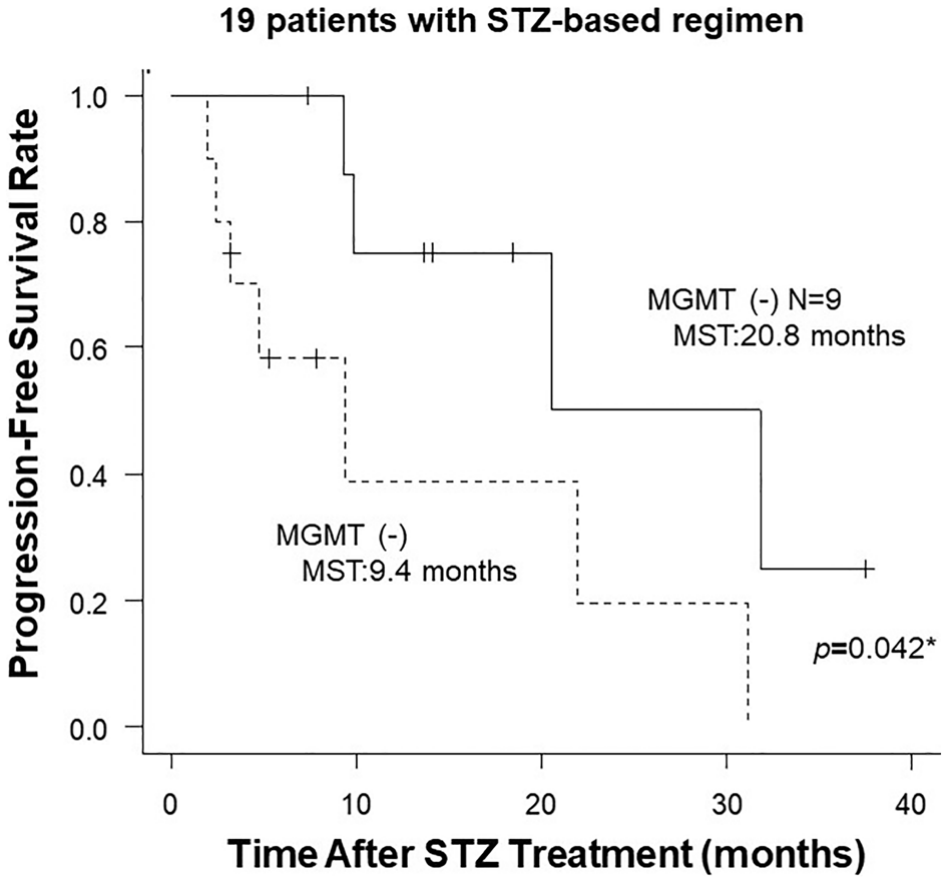
Progression-free survival from the start of STZ treatment in patients with positive and negative MGMT expression. Statistical significance was determined by log-rank test.

Regarding the relationship between MGMT expression and prognosis according to progression-free survival (PFS), Kaplan–Meier analysis revealed that positive MGMT expression was significantly associated with a worse prognosis for STZ-based treatment regimens (*p* = 0.042) (Fig. 5). The median PFS was 20.8 months in MGMT-negative patients and 9.4 months in MGMT-positive patients.

## Discussion

Higher-grade pancreatic neuroendocrine neoplasms are considered to have a poorer prognosis than low-grade neoplasms, due to the faster growth rate of tumor cells and the potential for developing liver metastasis^27,28^. In this study, we demonstrated that the tumor grade was significantly higher in MGMT-negative tumors (*p* < 0.001). In particular, MGMT negativity was significantly more frequent in NET-G2 (35/56 cases, 62.5%) than in NET-G1 (9/80 cases, 11.2%, *p* < 0.001, Table 1). In addition, we also demonstrated that Pan-NET with negative MGMT expression exhibited significantly favorable therapeutic efficacy of STZ-based treatment (Figs. 3, 4) and a better prognosis after STZ-based treatment (Fig. 5).

Previously, STZ was considered to have high therapeutic efficacy against tumors with a high Ki67 index greater than 5%^7^. However, the underlying biological mechanism had not been clarified. In this study, we provided evidence that STZ therapy was more effective in NET-G2 than in NET-G1, since MGMT inactivation was frequently increased in NET-G2.

TMZ and STZ are mainly classified as alkylating agents in terms of their mechanism of action. Alkylating agents function as cytotoxic anticancer agents and play a crucial role in the treatment for advanced Pan-NET^6^. MGMT is known to repair alkylating agent damage to malignant tumors and inhibit the effects of alkylating agents. The relationship between the therapeutic effects of alkylating agents and MGMT activity has been reported, especially in treatment with TMZ, and a consensus has been established regarding the treatment of brain tumors^12–14^. Knowledge of the therapeutic effects of TMZ on Pan-NET associated with MGMT activity has gradually developed^16–25^.

However, there are only a few reports focusing on the relationship between STZ and MGMT activity in Pan-NET^19,25,26^. Walter et al. examined MGMT activity in 20 cases of NENs, including gastrointestinal NENs and pulmonary NENs, and reported a negative correlation between MGMT activity and the treatment effect of STZ^19^. Krug et al.^24^ examined MGMT activity in 24 NENs, including gastrointestinal NENs and pulmonary NENs, and concluded that MGMT activity is not a prognostic predictor for STZ treatment outcomes. These 2 reports include many NENs other than Pan-NET, making it difficult to make a simple association between MGMT activity and the treatment effect of STZ in Pan-NET. Hijioka et al.^26^ examined 13 cases of Pan-NET and reported that MGMT could be a predictor of the treatment response to STZ; however, the study did not address the prognosis. In addition, none of the reports clarified the relationship between MGMT activity and the ki-67 index in Pan-NET. Thus, the relationship between MGMT and the therapeutic efficacy of STZ has remained controversial.

Although TMZ and STZ have similar mechanisms of action, there are no published studies comparing their clinical efficacy, making it difficult to identify the more effective therapy. In MGMT-negative tumors, an additional therapeutic effect can be expected if there is a certain withdrawal period between the administration of two different alkylating agents. This sequential strategy can be applied for CAPTEM followed by STZ-based regimens or STZ-based regimens followed by CAPTEM for Pan-NET, a rare disease with few treatment options^29^. However, concerns about increased MGMT expression after treatment with an alkylating agent should also be considered^29^.

In this study, we comprehensively examined MGMT expression levels in various grades of surgically resected Pan-NET by IHC staining, and clearly demonstrated a significant difference in patient prognosis after STZ treatment between MGMT-negative and MGMT-positive expression. In glioblastoma, IHC is often used to analyze MGMT protein expression, and MGMT levels are assessed by the percentage of MGMT-positive cells in the nucleus. The most frequently used cutoff is 10%, as applied in this study. Cutoffs of 5–35% have been used to assess positive MGMT protein expression in glioblastoma^15^. MGMT negativity might be a companion marker of favorable therapeutic efficacy for STZ. There is a need to generalize the method of evaluating the MGMT status to permit its clinical application.

MGMT expression should be evaluated in metastatic sites if possible. It has been reported that MGMT methylation is increased in liver metastases in colorectal cancer^30^, suggesting that MGMT expression is decreased in liver metastases. It might be important to evaluate MGMT expression in metastatic sites prior to treatment.

In addition, the association between MGMT expression and therapeutic efficacy in other therapeutic agents was also evaluated. Sunitinib is often used clinically in an advanced setting of Pan-NET as well as STZ. When the association between the treatment response and MGMT expression was examined in 34 patients receiving sunitinib treatment, there was no significant difference of PFS (Supplementary Fig. 1). This result supports that MGMT expression is specifically associated with therapeutic efficacy in STZ treatment.

MGMT is associated with DNA repair signaling, which is often impaired in cancer cells^31^. Inhibition of MGMT expression is known to be associated with genomic instability^32^. Furthermore, in this study, we found that the frequency of MGMT expression was decreased in higher grade Pan-NET, suggesting that MGMT may be associated with a tumor suppressive effect in Pan-NET. BRCA1, similar to MGMT, also functions as a tumor suppressor gene and is involved in DNA repair signaling. PARP inhibitors are DNA damage-inducing anticancer agents, like STZ. The association between MGMT and the therapeutic effect of STZ in Pan-NET may be analogous to the relationship between BRCA1 and the effect of PARP inhibitors. Thus, this is a basic therapeutic concept for anticancer drugs based on mutation status in specific DNA repair-related genes such as MGMT and BRCA1^33^.

There were several limitations in this study. It was a single-center analysis. In addition, the retrospective nature of the study may have made it prone to selection bias. A multicenter study is recommended in the future.

Furthermore, the 19 patients treated with STZ in this study are likely to represent at heterogeneous group because this population consists of Pan-NET of various grades treated with different regimens. Surgical samples were obtained after STZ treatment in some patients. The use of multiple agents rather than a single agent might affect MGMT expression and STZ efficacy in terms of pharmacologic interference. However, concerning the treatment regimens, it is difficult to measure the effect of individual agents because combination therapy is commonly used in clinical practice. This is one of the limitations of this study, and further case accumulation is needed.

In this study, we examined 146 Pan-NENs cases including 19 STZ-treated cases by IHC staining and reported the MGMT expression profile by tumor grade in Pan-NET. We reported that the Ki-67 index and MGMT protein expression are negatively associated in Pan-NET. MGMT negativity was significantly more frequent in NET-G2 than in NET-G1. We also demonstrate that PFS in MGMT-negative cases was significantly better than in MGMT-positive cases in patients treated with STZ. Taken together, the reduced expression of MGMT in NET-G2 is anticipated to confer a better therapeutic effect on STZ-based regimens than observed in NET-G1.

## Conclusion

The MGMT expression level can be a good indicator for determining the efficacy of STZ-based treatment for Pan-NET. The frequency of MGMT expression is lower in NET-G2 than in NET-G1, indicating that it may be a companion diagnosis to estimate the therapeutic efficacy of STZ-based treatment, especially in patients with NET-G2.

## Supplementary Information

Supplementary Figure 1.

Supplementary Table 2.

Supplementary Table 3.

Supplementary Table 4.

## Data Availability

Data generated or analysed in Figs. 2 and 5, and Supplementary Table 1 during this studyare included in this published article and its supplementary information files. Otherwise, all datasets used and/or analysed during the current study available from the corresponding author on reasonable request.

## Abbreviations

Pan-NENs: Pancreatic neuroendocrine neoplasms
Pan-NET: Pancreatic neuroendocrine tumor
MiNEN: Mixed neuroendocrine-non-neuroendocrine neoplasm
IHC: Immunohistochemistry
MGMT: O6-methylguanine DNA methyltransferase
STZ: Streptozocin
PD: Progressive disease
SD: Stable disease
PR: Partial response
