# Supplementary figures and images for: MGMT is frequently inactivated in pancreatic NET-G2 and is associated with the therapeutic activity of STZ-based regimens
Source: Sci Rep. 2023 May 9;13:7535. doi: 10.1038/s41598-023-34666-y (PMC10170117; doi:10.1038/s41598-023-34666-y)

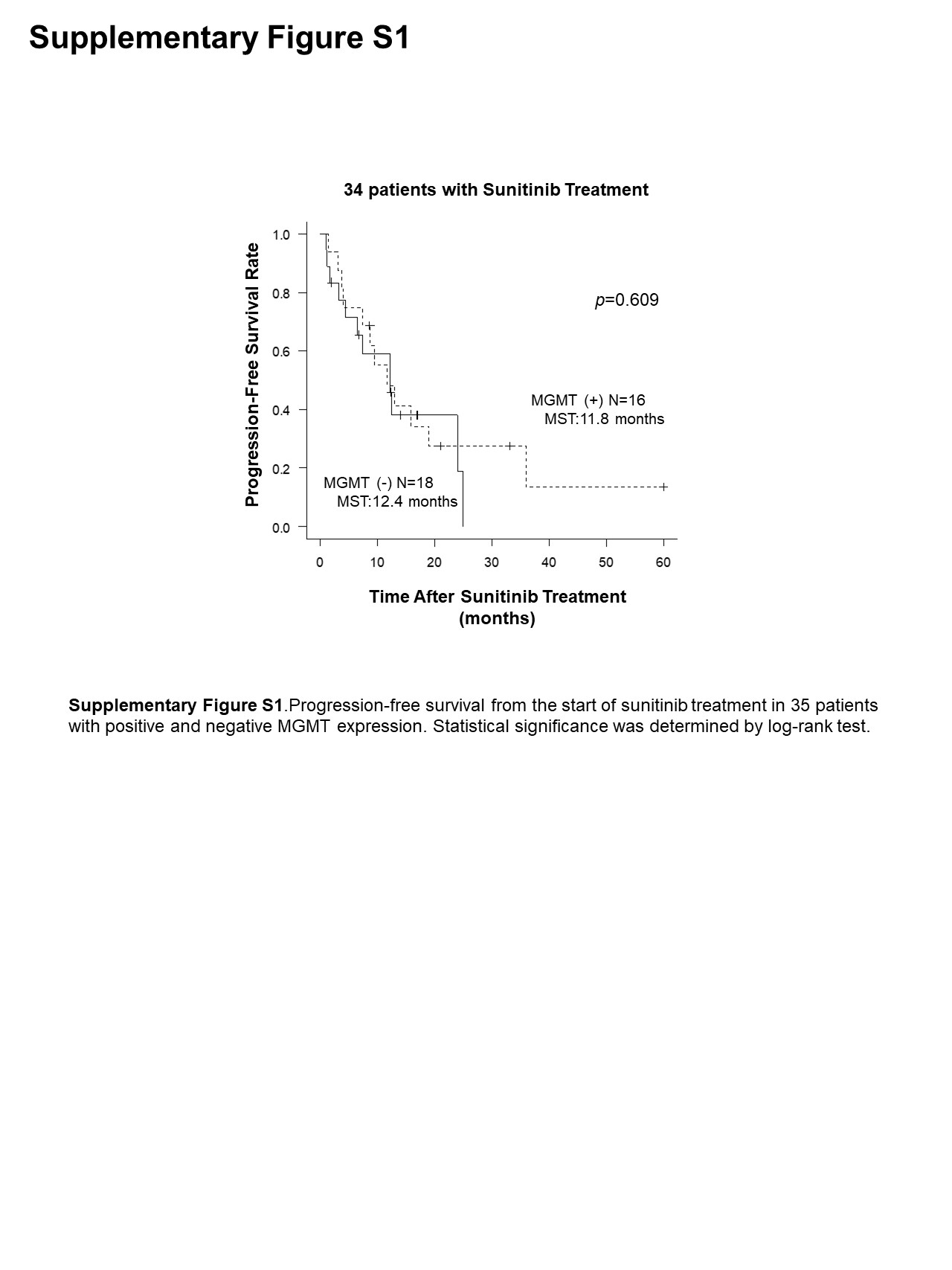

Supplement: Supplementary file 1 — Supplementary Figure 1. [file 41598_2023_34666_MOESM1_ESM.jpg]
