# Supplementary material for: MGMT is frequently inactivated in pancreatic NET-G2 and is associated with the therapeutic activity of STZ-based regimens
Source: Sci Rep. 2023 May 9;13:7535. doi: 10.1038/s41598-023-34666-y (PMC10170117; doi:10.1038/s41598-023-34666-y)
Supplement: Supplementary file 2 — Supplementary Table 2. [file 41598_2023_34666_MOESM2_ESM.docx]

**Supplementary Table1.** Univariate and multivariate analysis of overall survival in 142 Pan-NEN patients

| Clinicopathological factor | Univariate analysis |  | Multivariate analysis |  |  |
| --- | --- | --- | --- | --- | --- |
|  | HR (95% CI) | P value | HR (95% CI) | P value |  |
| Clinical factor |  |  |  |  |  |
| Age, ≥60 years | 0.8 (0.3 – 2.5) | 0.74 |  |  |  |
| Sex, male | 1.8 (0.6 – 5.4) | 0.31 |  |  |  |
| Lymph node metastasis (+) | 4.8 (1.6 – 14.4) | <0.006* | 2.8 (0.9 – 9.3) | 0.09 |  |
| Liver metastasis (+) | 3.8 (1.2 – 11.7) | <0.02* | 1.7 (0.5 – 5.6) | 0.4 |  |
| Functionality, nonfunctioning | 0.4 (0.04 – 2.7) | 0.31 |  |  |  |
| Genetic syndrome, n (%) |  |  |  |  |  |
| MEN type 1 | 1.5 (0.2 – 11.5) | 0.71 |  |  |  |
| VHL | - | 1 |  |  |  |
| Tumor factor |  |  |  |  |  |
| Tumor size, ≥17mm, median | 3.4 (0.94 – 12.4) | 0.062 |  |  |  |
| Ki-67 index, ≥2%, median | 3.5 (0.96 – 12.7) | 0.058 |  |  |  |
| Mitosis, ≥2 per 10 HPF, median | 7.6 (2.5 – 23.5) | <0.001* | 5.4 (1.7 – 18) | 0.005* |  |
| Chromogranin A (+) | 0.4 (0.1 – 1.6) | 0.2 |  |  |  |
| Synaptophysin (+) | - | 1 |  |  |  |
| CD-56 (+) | - | 1 |  |  |  |
| MGMT (+) | 0.6 (0.2 – 1.8) | 0.4 |  |  |  |
| *CI,* confidence interval; *HPF,* high-power fields; *MEN,* multiple endocrine neoplasia; *MGMT,* O6-methylguanine DNA methyltransferase; *VHL,* von Hippel–Lindau disease | | | | |  |
|  |  |  |  |  |  |
| *P* < 0.05 is considered significant |  |  |  |  |  |
